# Poisoning by Santonin

**Published:** 1877-11

**Authors:** 


Poisoning by Santonin.—Prof. Binz, of Bonn, has just recorded an interesting and important case of poisoning by santonin, which came under his observation two years ago. The patient was a delicate child, twenty-five months old, who, about 6 a.m., took two lozenges, each containing .025 miligramme of this drug. At 4 p.m., without any previous warning, sudden clonic convulsive spasms set in, commencing at the left angle of the mouth, and thence spreading over the left side of the face. These were succeeded by similar spasms in the right arm, beginning in the fingers. A quarter of an hour after, a tonic spasm invaded the left side of the face and left arm, then rapidly disappeared, leaving a fibrillary twitching of the muscles of the left angle of the mouth and left eyelid, which soon afterwards ceased quite suddenly. Two more convulsive attacks occurred on the same evening, and in one the respiratory movements threatened to come to a standstill, although the heart was beating quite strongly and the pulse was normal. Two or three similar fits occurred daily at intervals for the next four or five days, after which the child was as well as before. During this time the urine was of the peculiar greenish-yellow color which has been noticed after the exhibition of this drug. Prefesssor Binz suggests that the localization of the convulsive spasms on the left side of the face was due to its being smaller and less developed than the right side, thus "making it a place of less resistance." As santonin is not included among poisons in works on forensic medicine, the best treatment of such a case is a question of much importance, and to decide this, as well as its physiological action, some experimental investigations were made on cats, rabbits and frogs. Professor Binz injected weak solutions of santonate of soda in water, and found that the mesocephalon was first attacked, and that the convulsions principally affected the muscles supplied by the third, fourth, fifth, sixth and seventh cranial nerves. The spinal cord and the general muscular system were afterwards involved. There was no disturbance of the hearts action or of the blood pressure. Numerous observers have already described the peculiar effect of santonin on vision, causing objects to appear yellow and green. Nitrate amyl, morphia and artificial respiration, were powerless against the spasms, although the latter must be employed to ward off asphyxia from paralysis of the diaphragm. Ether and chloroform arrested the attacks if given at their very commencement, and always lessened their severity and duration. Hypodermic injections of chloral hydrate had much the same effect, so that we have in these agents very powerful physiological antidotes against the action of the poison, whilst we wait for its elimination.

The extremely prevalent use of santonin as a vermicide by the profession, and its common sale in worm powders and worm lozenges by every druggist, made the above case and observations of more than ordinary significance. Its occasional toxic action on the cerebrum has been noticed before, aud referred to by several writers ; but that it could induce such severe symptoms was not generally known, and it behooves us to give the drug with more precision, and watch its effects with more care, than has been the usual custom. The quantity taken in this instance—viz., a mere fraction of a grain—seems very small to have produced such a serious result; but as the child has never suffered from any convulsive symptom before or since, and as the urine was so evidently colored by the drug, we must either suppose that there was an unusual susceptibility to its action, or, more probably, that by some mistake the dose was much larger than was inferred from the lozenges that were analyzed. The effects on the lower animals prove its toxic power, and leave no doubt as to the necessity of our using the remedy much more sparingly than we do at present; and we are at least bound to take some trouble to ascertain the probable presence of worms in a child's alimentary canal before administering a medicine which may occasionally even act as a poison.

